# Reported methods for handling missing change standard deviations in meta-analyses of exercise therapy interventions in patients with heart failure: A systematic review

**DOI:** 10.1371/journal.pone.0205952

**Published:** 2018-10-18

**Authors:** Melissa J. Pearson, Neil A. Smart

**Affiliations:** School of Science and Technology, University of New England, Armidale, New South Wales, Australia; Universita degli Studi di Ferrara, ITALY

## Abstract

**Background:**

Well-constructed systematic reviews and meta-analyses are key tools in evidenced-based healthcare. However, a common problem with performing a meta-analysis is missing data, such as standard deviations (SD). An increasing number of methods are utilised to calculate or impute missing SDs, allowing these studies to be included in analyses. The aim of this review was to investigate the methods reported and utilised for handling missing change SDs in meta-analyses, using the topic of exercise therapy in heart failure patients as a model.

**Methods:**

A systematic search of PubMed, EMBASE and Cochrane Library from 1 January 2014 to 31st March 2018 was conducted for meta-analyses of exercise based trials in heart failure. Studies were eligible to be included if they performed a meta-analysis of change in exercise capacity in heart failure patients after a training intervention.

**Results:**

Twenty two publications performed a meta-analysis on the effect of exercise therapy on exercise capacity in heart failure patients. Eleven (50%) publications did not directly report the approach for dealing with missing change SDs. Approaches reported and utilised to deal with missing change SDs included imputation, actual and approximate algebraic recalculation using study level summary statistics and exclusion of studies.

**Conclusion:**

Change SDs are often not reported in trial papers and while in the first instance meta-analysts should attempt to obtain missing data from trial authors, this information is frequently not forthcoming. Meta-analysts are then forced to make a decision on how these trials and missing data are to be handled. Whilst not one approach is favoured for dealing with this matter, authors need to clearly report the approach to be utilised for missing change SDs. Where change SDs are imputed meta-analyst are encouraged to explore several options and have a sound rationale as to the choice, and where data is imputed, sensitivity analysis should be conducted.

## Introduction

Systematic reviews (SRs) and meta-analyses (MAs) serve key purposes; identifying, synthesizing and critically reviewing evidence, answering a specific question[1, 2]. Well-constructed SRs and MAs play a key role in evidenced-based healthcare helping inform clinical guidelines and practice[3, 4]. Furthermore, and as importantly, they assist in identifying knowledge gaps and research needs[4, 5]. When feasible, systematic reviews use meta-analysis, the statistical method for combining two or more studies to provide an estimate of the overall effect[2].

For meta-analysis of continuous variables, the standard approach requires information on the mean, standard deviation (SD) or standard error (SE) and sample size, in order to calculate an effect size[6]. There are multiple ways to calculate the effect size including change score from baseline and follow-up scores. However, a common situation that arises when the change score method is utilised is that change SD may not be reported[7]. While the best approach is to obtain any missing data from the original study authors, this is not always feasible or possible. The absence of and inability to obtain data from authors may result in the omission of studies from the review and analysis. However, omission of studies from a meta-analysis may reduce statistical power and potentially cause bias[7]. For this reason, meta-analysts utilise a range of methods to estimate SDs [6–8].

The Cochrane Handbook provides guidelines on a number of methods that can be utilised to calculate missing change SDs[6]. Reported summary statistics such as confidence intervals (CIs), t-values and p-values can be used for algebraic recalculation of SDs[6]. In instances when exact levels of significance are not reported, but significance is represented by an upper limit, i.e., p<0.05, then a conservative approach using the limit provided is often utilised[6]. Where reported data does not allow for algebraic recalculation, SDs may be imputed[6]. Both single study level imputation and multiple imputation methods exist. Of the simpler single study level imputation, common methods include assuming a relationship between the baseline SD and post intervention SD, and utilising this correlation to impute a change SD, and the use of direct substitution with a baseline or post-intervention SD utilised as the change SD[7]. Recently, fifteen methods were identified for dealing with missing SDs in meta-analyses[8]. This is in addition to the methods previously identified by Wiebe and colleagues in 2006[7]. An in-depth review of available methods to deal with missing SDs and variance data is beyond the scope of this review and readers are referred to the reviews by Weir et al. (2018)[8] and Wiebe et al. (2016)[7].

Heart failure remains a leading cause of morbidity and mortality worldwide. One of the primary symptoms of heart failure is reduced exercise tolerance. As cardiorespiratory fitness is linked to heart failure prognosis [9, 10], therapies that improve exercise capacity are of interest to clinicians, patients and researchers. Results of primary research and secondary level research via SRs and MAs of exercise therapy in heart failure, have led to exercise therapy being a Class 1A recommendation for stable heart failure patients, due in part to its ability to improve exercise tolerance[11]. Therefore, changes in exercise capacity from baseline to post intervention represent a continuous outcome frequently measured and analysed in this population and therefore is a suitable model upon which to investigate methodological approaches utilised. The primary aim of this review was to investigate what approaches were reported by meta-analysts for handling missing change SDs in the analysis of exercise capacity after exercise therapy interventions in heart failure patients.

## Methods

### Search strategy

Potential studies were identified by conducting systematic searches of PubMed, EMBASE, and the Cochrane Library from 1^st^ January 2014 to 31^st^ March 2018. Search terms were pre-specified. The following search terms were utilised to search databases: “heart failure”, “exercise”, “aerobic exercise”, “resistance training”, “yoga”, “tai chi”, “functional electrical stimulation”, “inspiratory muscle training”, “hydrotherapy”, “physical activity”, “physiotherapy”, “Kinesiotherapy”, “cardiac rehabilitation”, and “exercise therapy”. The search was limited to systematic reviews and meta-analyses published in English from 2014 to 2018. The search strategy is presented in S1 Appendix. One reviewer (MJP) conducted the search and two reviewers (MJP and NAS) screened the titles and abstracts of identified citations using pre-specified eligibility criteria. Potentially relevant publications were then fully reviewed to identify studies for inclusion.

### Inclusion criteria

Publications were included if they were published in English between 1st January 2014 and 31^st^ March, 2018. The search period was limited as there are numerous meta-analyses in this field and new methods have been introduced, so we wished to provide a contemporary approach. Meta-analyses were included if they analysed continuous variables of either VO_2peak_, 6MWT, peak power, exercise time or combined exercise capacity, in heart failure patients undertaking exercise training. As the issue of missing SDs is generally more of a problem when the MA is conducted using the mean difference or standardised mean difference for change (pre-post intervention) between an intervention and control group, for the purpose of this review we only included MAs that conducted statistical analysis using the change score method; hence MAs that utilised follow-up methods for analysis were excluded. Exercise training included any form of exercise therapy, including physical therapy modalities of functional electrical stimulation and inspiratory muscle training. Meta-analyses included studies of any design. Meta-analyses in which other interventions were analysed, i.e., pharmacological therapy or diet, were only included if exercise was analysed separately. Publications which identified themselves as a meta-regression analysis were included only if they provided the methods, results and details of the associated meta-analysis.

### Exclusion criteria

Meta-analyses were excluded if they included populations in addition to heart failure patients, even if heart failure patients were included and a separate analysis and/or a sub analysis were provided on heart failure patients. Abstracts or articles not available as full-text and non-English publications were excluded. Meta-analyses were excluded if they did not analyse the specified outcomes.

### Data extraction

Data extraction was performed by one reviewer (MJP). For each meta-analysis the following information was extracted: 1) author, year of publication and Journal, 2) study designs included in analyses, 3) type of intervention, 4) exercise capacity outcome measure, 5) statistical methods applied for meta-analysis, and 6) details of methods reported and utilised to handle missing SDs.

### Data analysis

The primary outcome to be assessed in the review was identification of the reported approach in meta-analyses for dealing with missing change SDs. Meta-analyses, including supplementary files were individually reviewed and categorised based on the approach reported in the publications to handle missing change SDs. Meta-analyses were allocated to one of four categories 1) No clear approach reported, 2) Algebraic or approximate algebraic recalculation of SD, 3) Imputed SD, and 4) No imputation (studies with unrecoverable missing change SDs were excluded from the meta-analysis). Where studies reported using more than one method for dealing with missing change SDs (e.g., use of algebraic calculation and imputation) they were allocated to both categories in the results. Where no approach for handling missing change SDs was reported, our secondary aim was to examine, where possible, a random selection of studies in the identified meta-analysis in an attempt to ascertain what approach may have been adopted by the meta-analyst.

## Results

**Fig 1:**
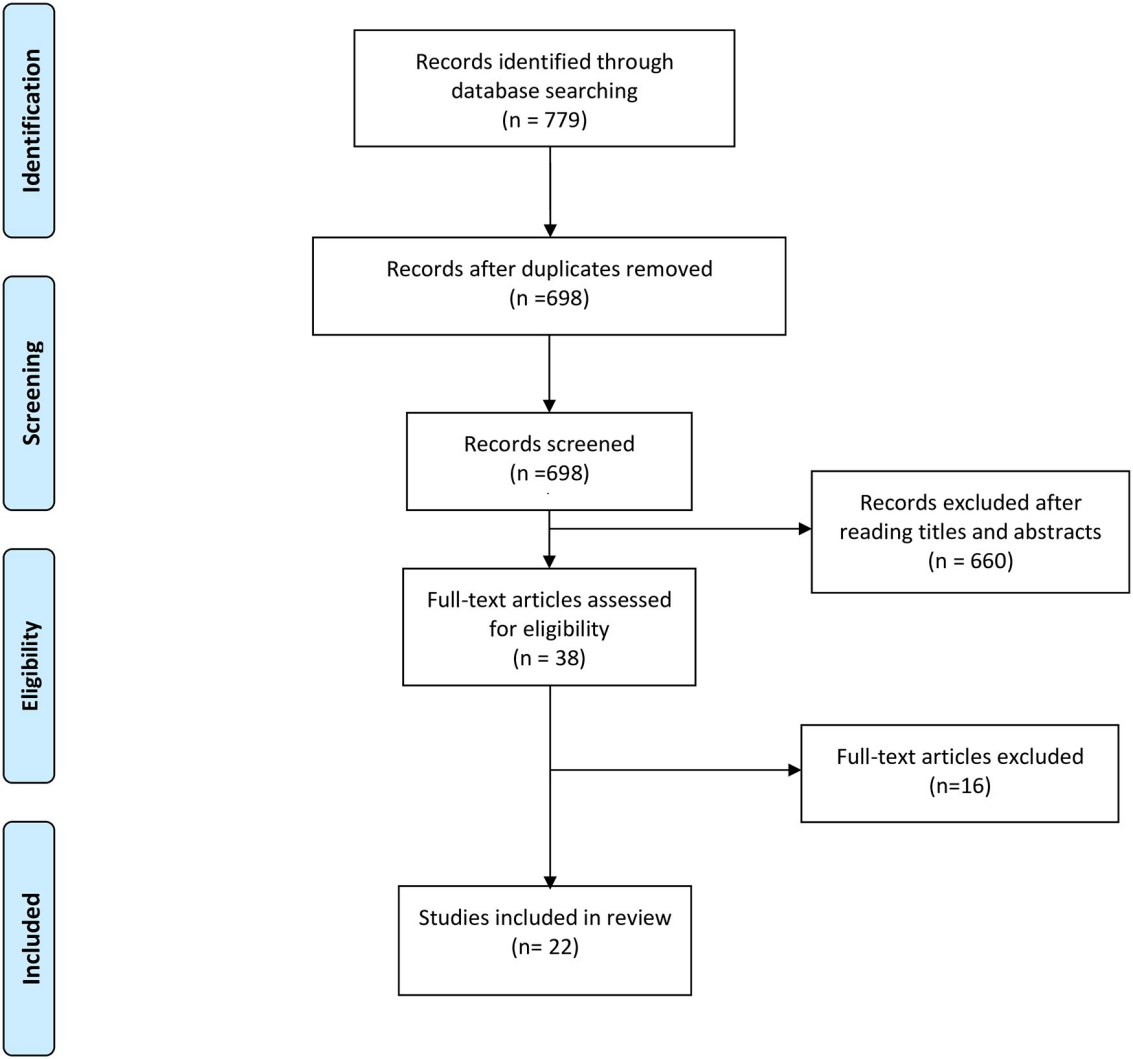
PRISMA Study flow diagram.

The initial search generated a total of 779 articles. After removal of duplicates and exclusion of articles based on abstract and title, 38 full-text articles remained for screening. Full screening resulted in 22[12–33] articles meeting the stated inclusion criteria (Fig 1). Details of MAs reviewed, but excluded are provided in S1 Table.

### Characteristics of included meta-analyses

A general description of included analyses is provided in Table 1. Twenty two [12–33] MAs reported on change in exercise capacity. Meta-analysis of VO_2peak_ was performed in 21 publications[12–16, 18–33], 6MWD was analysed in 12 publications[12–17, 19, 20, 22, 25, 28, 33] peak power in two[12, 21] and exercise time in one[26] publication. Analyses examined a range of exercise modalities across heart failure phenotypes, and Review Manager (Revman) was the most popular software package utilised. Additional details of included publications are reported in S2 and S3 Tables. The publications were spread across 14 journals.

**Table 1:** Summary of characteristics of meta-analyses included in review.

Characteristics	Number
Meta-analyses included in review	22
Associated Journals	14
Study Designs included in Meta-analysis	
• RCTs only	18
• RCTs & Controlled Trials	4
Year Meta-analysis published	
• 2014	4
• 2015	4
• 2016	7
• 2017	4
• 2018	3
Exercise Training/Therapy Modalities included in MAs	
• Aerobic only	3
• Resistance only	2
• Aerobic, Resistance and/or Combined Training	3
• Mixed modalities (aerobic, resistance, combined, yoga, tai chi, NMES and/or IMT)	6
• Tai Chi only	2
• Yoga only	1
• Hydrotherapy/Aquatic only	2
• IMT only	1
• NMES and other Exercise	2
Exercise Capacity Outcome Measures in MAs	
• VO_2peak_	21
• 6MWT	12
• Peak Power/Workload	2
• Exercise Time	1

6MWT: 6-minute walk test, IMT: inspiratory muscle training, MAs: meta-analysis, NMES: neuromuscular electrical stimulation, RCTs: randomised controlled trials

### Reported approaches for handling missing change SDs

A number of publications note contacting authors for missing and raw data; however, no MA reported the specific details on any SD data requested, and if the requested information was provided. A number of MAs within the review reported in the methods that more than one approach for dealing with missing change SDs would be adopted if required. Specifically, two MAs [24] [28] reported that algebraic recalculation from summary statistics (i.e, t-values or 95%CI) would be used, an in addition data presented as median (IQR) would be converted. Only one MA [21] reported that a correlation coefficient would be used where possible, but if no data was available for this calculation then the study would be excluded. Only one MA quantified the number of studies with missing SDs and how each of these studies was dealt with[21]. However, this was specifically in regard to the pre and/or post SD required to calculate a change SD[21]. Two MAs [12, 29] report the exclusion of studies if suitable data was not available, however, this appears to be only in the case where both mean and SD were missing. Only one MA[21], specifically reported excluding a study due to missing pre/post SD required to impute a change SD, and no other MA utilising change values for meta-analysis made reference to the possibility of missing pre or post SDs and how this would be handled. Table 2 provides a summary of approaches as reported in the MAs to deal with missing change SDs.

**Table 2:** Summary of approaches to deal with missing change SDs as reported in the meta-analyses.

Approach	Method reported in Publication	Number of Publications
No details reported/unclear	No clear method for handling missing change SDs reported in the paper	11
Algebraic or approximate algebraic recalculation	SD calculated using SE, t or F- statistics, 95% CI, actual p-values or default p-values using upper or lower bound p-values and non-significant p-values	6
	Data extracted from figures/visual analysis	1
Study level imputation	Imputation using a correlation value	4
	Directly substituted SD–Baseline SD used as change SD	1
	Imputation of mean±SD using median and interquartile range (IQR)	4
No Imputation	Studies excluded from meta-analysis if data not available to impute a SD	1

CI: confidence interval, SD: standard deviation, SE: standard error

#### 1) No method of handling missing change SD reported

Overall eleven [12, 17, 22, 23, 25–27, 29, 31–33] MAs failed to report anywhere within the publication any clear approach to deal with missing change SDs. Further examination of the 11 MAs revealed a range of methods were utilised in these analyses, including actual and approximate algebraic recalculations using actual or default p-values, post-intervention SDs and imputed SDs using correlation values (Table 3).

**Table 3:** Summary of approaches utilised to deal with missing change SDs from the 11 publications where no approach was reported.

Approach	Method utilised	Number of Publications
Approach still unclear	Approach still unclear after examination of a random selection of individual studies included in MAs	5
Algebraic or approximate algebraic recalculation	SD calculated using SE, t or F- statistics, 95% CI, actual p-values or default p-values using upper or lower bound p-values and non-significant p-values	1
Study level imputation	Imputation using a correlation value	2
	Directly substituted SD–Follow-up SD used as change SD	4

CI: confidence interval, SD: standard deviation, SE: standard error

#### 2) Algebraic or approximate algebraic recalculation of SD

One MA[28] noted that the *t-* value was utilised to calculate SD change. Five MAs [13, 14, 18, 19, 24] report utilising CIs, actual p-values or default p-values when an exact p-value was not available. Hence, in the case of default p-values the SD value becomes an approximate algebraic recalculation. Further individual examination of 4 MAs [13, 14, 18, 19] that noted using actual or approximate p-values revealed that both actual and default p-values were utilised. Further review of one MAs[24] indicated that while the CI was reported as being utilised, the post intervention SD was directly substituted for the change SD in the analysis. One[21] MA noted the extraction of data from one included study using visual analysis.

#### 3) Imputation of SD

If baseline and follow-up SDs are known, it is possible to calculate a missing change SD using a value that represents the correlation between baseline and follow-up scores[6]. If the actual correlation coefficient is not reported this value can be imputed[6]. Three MAs[15, 16, 30] reported utilising a specific correlation value to calculate the change SD. Values of 0.8[16], 0.5[15] and 0.7[30] were utilised, however, only the study that utilised 0.5 noted why, stipulating it was a conservative value[15]. One additional MA[21] noted using this same method to calculate the SD of each study; however, the correlation value was not reported. Direct substitution was only reported in one MA[20], with the baseline SD utilised. Four MAs [17, 24, 28, 29] noted the conversion of median and interquartile (IQR) to mean±SD. Of these, one[17] calculated the SD using IQR/1.35, one[24] stipulated using previously established methods, referencing Wan et al. (2014)[34], one[29] utilised a formula provided in the paper (S2 Table), while one MA[28] notes using a formula, but provided no details. No MAs reported the use of multiple imputation methods.

#### 4) No imputation

One MA[21] reported the exclusion of studies if SDs were not available to calculate the change score SD.

#### Sensitivity analysis

No meta-analysis reported performing a sensitivity analysis specifically in relation to imputed SDs in order to examine the impact of different imputed values. Three MAs[14, 18, 19] that adopted the approach of utilising default p-values when actual values were not available, did however note that this approach was a limitation, which could “introduce errors” into the analysis.

#### The problem of missing change SD and reporting of methods

While it is possible that authors do not report on a method for dealing with missing change SDs due to the fact that no change SDs are missing for included studies, it has and continues to be consistently reported over time that missing change SDs are a problem. In an attempt to understand and highlight the nature of the problem over time, we conducted a review of three meta-analyses (based on the eligibility criteria for this review) from three different time periods. In 2004 the first Cochrane Review[35] of exercise-based rehabilitation in heart failure patients conducted a statistical analysis of VO_2peak_ and 6MWT. The review does report a method for dealing with missing change SDs, outlining the following method: *“Where standard deviation differences were not reported in the source papers*, *allowance has been made for within patient correlation from baseline to follow-up measurements by using the correlation coefficient between the two (Cochrane Heart Group; Follman 1992)”*. After conducting a review of the eight studies included in the analysis of the 6MWT, it is clear that the actual change mean±SD (or SE) was only reported in the source publications of three studies and this data subsequently utilised in the analysis. Based on a review of the publications of the remaining five included studies, it appears that only the pre and post intervention data were reported with no change data, hence the change SD has been imputed in the meta-analysis. No specific details of the exact correlation coefficient(s) utilised are provided, however, a brief audit of these studies indicates that a correlation value of 0.5, which is considered a conservative value, may have been utilised for these studies. Although 2004 is the first time, we aware of, that imputation techniques have been used, they did not become part of mainstream practice until about 10 years later.

In a 2013 meta-analysis Smart and colleagues[36] examined the change in VO_2peak_ in functional electrical stimulation compared to cycling; five studies were included in the analysis. However, no trial included in the meta-analysis reported the change mean±SD in the published trial paper. The meta-analysis was performed using the change between pre and post intervention means for each intervention group and the change SD calculated, as described by the authors in the methods: *“Data used were continuous and were reported as mean and standard deviation*. *Revman 5*.*1 enabled calculation of post-intervention change from baseline for standard deviation*, *using the change in mean values*, *number of subjects and p value for each group”*. A review of the studies included in this meta-analysis indicated that only one of the included studies (for VO_2peak_ FES vs. cycling) reported the actual p values, from which the change SD was calculated. The change SD for the remaining four studies was imputed using upper/lower limits of estimated p-values, e.g., if p<0.05, then p = 0.049 was utilised, if p<0.001, then p = 0.00099, if p = not significant (ns) then p = 0.051 was utilised.

The above two analyses highlight the issue of missing SDs and the issue of missing change SDs has continued. A recent large meta-analysis by Santos et al. (2018)[29] included 46 data sets (from 41 studies) in the analysis of VO_2peak_. However, a review of the studies included in the analysis indicated that only seven studies reported the change mean±SD (or SE) in the published paper, with all remaining studies only reporting pre and post intervention data. While not stated within the methods of this meta-analysis, a review of data indicates that in conducting the analysis the authors’ imputed all change SDs for all included data sets, utilising a correlation coefficient of 0.0.

While both the 2004[35] and 2013[36] analyses above specifically reported a method within the paper to handle missing change SDs, a review of a random sample of meta-analyses between 2004 and 2014, revealed no consistency in meta-analysts reporting on a method to handle missing change SDs.

## Discussion

This review examined methods currently reported and utilised to handle missing change SDs in the statistical analysis of exercise capacity in heart failure patients after exercise therapy interventions. While all publications in the current review included details of the applied statistical methods for meta-analysis, reporting of the specific approach that would be employed to handle missing change SDs was absent in 50% of publications. The omission of an approach to deal with missing change SD is not to say that the approach or methods applied by the meta-analysts are in anyway contrary to what is recommended. However it does raise a number of issues when interpreting the results and drawing conclusions. Not only does the interpretation of MAs become difficult with absent or ambiguous information, but insufficient detail on methods and assumptions applied to handle missing change SDs impacts the transparency and reproducibility of the meta-analysis[37].

An increasing number of methods to dealing with missing variance data, including SDs are reported in the literature [7, 8, 38–40]. Of the MAs included in our review, use of algebraic or approximate algebraic recalculations using study summary statistics, was the most commonly reported approach. However, in the majority of cases, identification as to whether MAs utilised actual or approximate algebraic recalculations and which summary statistics, was only evident upon an individual examination of the studies included in the MAs.

Three imputation methods were reported in the reviewed publications; use of a correlation to calculate a change SD, direct substitution using an alternative SD (e.g., baseline or follow-up SD), and conversion of median (IQR) data. Missing change SDs can be calculated if pre and post SDs are available utilising an imputed value for the correlation coefficient[6]. The correlation value can be acquired from a number of sources, including using data from a similar study, from a similar meta-analysis, use of what is often considered a conservative value (0.5), or an assumption of no correlation (0.0), which is the most conservative[6, 7]. However, only one of the included MAs to adopt a correlation value provided a reason for the value. While median (IQR) data is actual, and not borrowed data it is also considered a method of imputation[7], and hence was included as an approach in our review. Interestingly, while four MAs made note of the conversion of median (IQR) in the methods when no mean±SD was provide, only two of these provided an approach to dealing with missing change SDs when mean data was reported.

Only one[21] MA identified the studies with missing change SDs and how different approaches were applied. The finding that the majority of the MAs did not specifically identify which studies had imputed SDs or whether SD data was acquired from authors, is in accordance with findings of Page and Colleagues (2018)[37] in their review of reproducibility of research practices in systematic reviews.

In the case of approximate algebraic recalculation or imputation, no MA reported performing sensitivity analysis in regard to these assumptions. While previous studies have suggested that different methods of imputation for missing variances do not alter the conclusion of MAs[41, 42], the results of these reviews in specific populations or involving specific interventions, cannot be generalised and applied to all MAs. Importantly, as imputation involves making assumptions, it is recommended that sensitivity analysis be performed to assess the impact of changing assumptions [6–8].

Given the large percentage of publications without a clear statement of approach to dealing with missing change SDs, and in an attempt to create a more accurate picture of the range of methods currently utilised, we reviewed a sample of individual studies included within the identified MAs. Upon further investigation we were able to determine the methods utilised by the majority of authors; which were consistent with the reported approaches. We did not attempt to completely reproduce any meta-analysis, but identify what methods may have been utilised in the case of missing change SDs. Interestingly, two studies[43, 44] not meeting the inclusion criteria for the review, due to the use of follow-up scores for statistical analysis, specifically noted the use of follow-up scores due to the lack of reporting of change SDs.

One of the reasons for performing a meta-analysis is to increase statistical power and provide context[2], and the omission of studies from a systematic review and meta-analysis due to missing SDs may result in bias and reduce the overall power[7]. If meta-analysts consider it inappropriate, for whatever reason, to calculate or impute missing SDs, it may be appropriate to present studies not included in the statistical analysis in a non-pooled tabulated format or provide an additional narrative review [6–8] so as not to exclude possible valuable information.

While individual studies are essential for providing data, with the increasing volume of studies, clinicians, researchers and policy makers have less time to sift through and make sense of this primary research. Systematic reviews and MAs therefore play a key role; however, a review is only as robust as the data supporting it; therefore rigor in design and reporting is crucial. A number of reporting guidelines exist to assist the meta-analyst, with the PRISMA Statement[3] one of the most frequently used. Designed to improve the transparency and reliability of systematic reviews and meta-analyses it states that authors should make note of how missing data was handled, any data transformations that are to be undertaken and which study results were not directly reported and required estimation[3]. In deciding on an approach to dealing with missing SDs, previously Wiebe and colleagues (2006)[7] provided a brief guideline, to which Weir et al. (2018)[8] suggest an additional step, and these guidelines represent a good starting point for the meta-analysts.

As best practice and in the first instance meta-analysts should attempt to contact trial authors for missing data. In the event that this information is not forthcoming, and as identified by Wiebe et al. (2006)[7] and Weir et al. (2018)[8], meta-analysts should proceed based on the information available. Where exact summary statistics are available they can be used for algebraic recalculation of the change SD, however, when these are not available the meta-analyst is left with the option of excluding studies or imputing the change SD, utilising multiple imputation, single level imputation and/or use of nonparametric statistics. In imputing the missing change SDs meta-analysts are encouraged to explore several options and have a sound rationale as to the choice. However, in any event sensitivity analysis should be conducted where data is imputed. Furthermore, where approximate algebraic recalculation and imputation are utilised, meta-analysts should clearly note these as possible limitations in that errors of varying sizes could have been introduced into the analysis.

The identification of fifteen new methods for handling missing SDs[8], in addition to the methods previously identified[7], highlight the expansion of statistical methodology applied in meta-analyses. There is no consensus as to which approach for dealing with missing SDs is best[39] and meta-analysts need to consider not only why the SD may be missing, but also the best approach in order to provide a comprehensive and robust presentation of the available evidence. Therefore, to improve the value of MAs, authors are encouraged to accurately report what they did and what they found. Meta-analysts should not only report the approach and methodology for handling missing change SDs in the methods section, or supplementary material, but also detail the number of studies with missing change SDs and identify the studies to which a particular approach has been applied. This can be done annotated in tables or supplementary files. All of which increase the transparency and reliability of the MA.

### Strengths and limitations in the systematic review

A limitation of this review was that it only considered MAs that measured exercise capacity as a continuous outcome, however, this is the most common continuous outcome reported to date in this population in regard to exercise training. Meta-analyses of other continuous outcomes may have reported and utilised additional methods to deal with missing change SD. Furthermore, the results of this review are only applicable to the population and intervention investigated. In studies in which no approach for handling missing change SDs was noted, we conducted a review of a sample of studies included in the MA in order to identify possible methods that had been applied.

## Conclusion

Systematic reviews and meta-analyses are a key component of evidenced-based healthcare and provide valuable information for researchers. Currently there is no one or standardised approach utilised in dealing with missing change SDs when assessing continuous outcomes and exercise interventions in heart failure. As a minimum meta-analysts should clearly stipulate how they will handle missing change SDs in the methods, and conduct sensitivity analysis when SDs are imputed.

## Supporting information

S1 Appendix Search strategy. (DOCX)

S1 Table Meta-analyses reviewed but excluded. (DOCX)

S2 Table Details of meta-analyses included in review. (DOCX)

S3 Table Additional details of meta-analyses included in review. (DOCX)

S4 Table PRISMA checklist. (DOC)
