# Supplementary material for: Reported methods for handling missing change standard deviations in meta-analyses of exercise therapy interventions in patients with heart failure: A systematic review
Source: PLoS One. 2018 Oct 18;13(10):e0205952. doi: 10.1371/journal.pone.0205952 (PMC6193694; doi:10.1371/journal.pone.0205952)
Supplement: S1 Appendix — (DOCX) [file pone.0205952.s001.docx]

**S1 Appendix - Search strategy**

**EMBASE**

Date Range 1.1.2014 – 31.3.2018

| Query No. | Query |
| --- | --- |
| 16 | #15 AND (2014:py OR 2015:py OR 2016:py OR 2017:py OR 2018:py) |
| 15 | #12 AND #13 AND ([cochrane review]/lim OR [systematic review]/lim OR [meta-analysis]/lim) AND [humans]/lim AND [english]/lim |
| 14 | #12 AND #13 |
| 13 | 'heart failure':ab,kw,ti |
| 12 | #1 OR #2 OR #3 OR #4 OR #5 OR #6 OR #7 OR #8 OR #9 OR #10 OR #11 |
| 11 | 'kinesiotherapy'/exp OR 'kinesiotherapy' |
| 10 | 'physiotherapy'/exp OR 'physiotherapy' |
| 9 | 'physical activity'/exp OR 'physical activity' |
| 8 | 'hydrotherapy'/exp OR 'hydrotherapy' |
| 7 | 'inspiratory muscle training'/exp OR 'inspiratory muscle training’ |
| 6 | 'functional electrical stimulation'/exp OR 'functional electrical stimulation' |
| 5 | 'tai chi'/exp OR 'tai chi' |
| 4 | 'yoga'/exp OR 'yoga' |
| 3 | 'resistance training'/exp OR 'resistance training’ |
| 2 | 'aerobic exercise'/exp OR 'aerobic exercise' |
| 1 | 'exercise'/exp OR 'exercise' |

**Cochrane Database of Systematic Reviews**

| Query No. | Query |
| --- | --- |
| 1 | (exercise):ti,ab,kw (Word variations have been searched) |
| 2 | ("cardiac rehabilitation"):ti,ab,kw (Word variations have been searched) |
| 3 | (yoga):kw (Word variations have been searched) |
| 4 | ("Tai Chi"):kw |
| 5 | (hydrotherapy):kw (Word variations have been searched) |
| 6 | ("inspiratory muscle training"):kw (Word variations have been searched) |
| 7 | ("functional electrical stimulation"):kw (Word variations have been searched) |
| 8 | ("neuromuscular electrical stimulation"):kw (Word variations have been searched) |
| 9 | ("physical activity"):kw (Word variations have been searched) |
| 10 | ("physiotherapy"):kw (Word variations have been searched) |
| 11 | #1 Or #2 Or # 4 Or #4 or #5 or #6 or #7 or #8 or #9 or #10 |
| 12 | ("heart failure"):ti,ab,kw (Word variations have been searched) |
| 13 | #11 AND #12 with Cochrane Library publication date between Jan 2014 and Mar 2018, in Cochrane Reviews |

**PubMed**

| Query No. | Query |
| --- | --- |
| 1 | Search "Exercise"[Mesh] |
| 2 | Search "Exercise Therapy"[Mesh] |
| 3 | Search "Exercise Movement Techniques"[Mesh] |
| 4 | Search "Cardiac Rehabilitation"[Mesh] |
| 5 | Search "Physical Therapy Modalities"[Mesh] |
| 6 | Search "Hydrotherapy"[Mesh] |
| 7 | Search "functional electrical stimulation"[Text Word] |
| 8 | Search "neuromuscular electrical stimulation"[Text Word] |
| 9 | Search "inspiratory muscle training"[Text Word] |
| 10 | Search #1 OR #22 OR #3 OR #4 OR #5 OR #6 OR #7 OR #8 OR #9 |
| 11 | Search "heart failure"[Title/Abstract] |
| 12 | Search (#30) AND #11 Filters: Meta-Analysis; Systematic Reviews; Review; Publication date from 2014/01/01 to 2018/03/31; Humans |
