# Supplementary material for: Reported methods for handling missing change standard deviations in meta-analyses of exercise therapy interventions in patients with heart failure: A systematic review
Source: PLoS One. 2018 Oct 18;13(10):e0205952. doi: 10.1371/journal.pone.0205952 (PMC6193694; doi:10.1371/journal.pone.0205952)
Supplement: S1 Table — (DOCX) [file pone.0205952.s002.docx]

**S1 Table - Meta-analyses reviewed but excluded**

| **Author** | **Reason for Exclusion** |
| --- | --- |
| Anderson 2017 | SR included other cardiovascular populations, as well as HF |
| Cipriano 2017 | No pooling of measure of exercise capacity in MA |
| Cornelis 2016 | Meta-analysis conducted using post-intervention mean±SD (i.e., follow-up scores) |
| Haddad 2017 | Meta-analysis conducted using post-intervention mean±SD (i.e., follow-up scores) |
| Lee 2017 | No pooling of measure of exercise capacity in MA |
| Lewinter 2015 | Meta-analysis conducted using post-intervention mean±SD (i.e., follow-up scores) |
| Ostman 2017 | No pooling of measure of exercise capacity in MA |
| Neto 2014 | Meta-analysis conducted using post-intervention mean±SD (i.e., follow-up scores) |
| Pandey 2017 | Patients with ICDs, these studies included HF and non-HF patients |
| Sties 2018 | SR only, no MA |
| Taylor 2014 | No pooling of measure of exercise capacity in SR |
| Taylor 2015 | SR included other cardiovascular populations, as well as HF |
| Tu 2018 | No pooling of measure of exercise capacity in MA |
| Uddin 2015 | Meta-analysis conducted using post-intervention mean±SD (i.e., follow-up scores) |
| Wu 2018 | No data pooling of 6MWT |
| Zwisler 2016 | Meta-analysis conducted using post-intervention mean±SD (i.e., follow-up scores) |
