# Supplementary material for: Reported methods for handling missing change standard deviations in meta-analyses of exercise therapy interventions in patients with heart failure: A systematic review
Source: PLoS One. 2018 Oct 18;13(10):e0205952. doi: 10.1371/journal.pone.0205952 (PMC6193694; doi:10.1371/journal.pone.0205952)
Supplement: S2 Table — (DOCX) [file pone.0205952.s003.docx]

**S2 Table** Meta-analyses included in review and details of methods for handling missing SD as reported in publications

| **Study** | **Exercise Intervention**  **Modality** | **Exercise Capacity Measure** | **Method for handling missing SD** |
| --- | --- | --- | --- |
| Adsett (2015) | Aquatic training | VO_2peak_, 6MWT, Peak Power | No clear details of methods for handling missing change SDs are reported. It was noted that a number of studies included in the SR were not included in MA due to insufficient raw data, but this appears to be when both mean and SD not available. |
| Chan (2016) | Exercise training  (included: aerobic, combined, IMT, FES) | VO_2peak_, 6MWT | Methods as stated in publication  *“Revman 5.1 enabled calculation of post-intervention change from baseline for standard deviation, using change in mean values, number of subjects and p value or preferably 95% confidence intervals. In many cases where exact p-values were not provided, we used default*  *values e.g. p<0.05 became p=0.049”*  No details are provided of the number of studies for which the change SD was calculated or the performance of any sensitivity analysis. |
| Dieberg (2015) | Exercise training  (included: aerobic, combined, IMT, FES) | VO_2peak_, 6MWT | Methods as stated in publication  *“Revman 5.1 enabled calculation of post-intervention change from baseline for SD, using change in mean values, number of subjects, and P value or preferably 95% CIs. In many cases in which*  *exact P values were not provided, we used default values e.g., P<0.05 became P = 0.049”*  No details are provided of the number of studies for which the change SD was calculated or performance of any sensitivity analysis. The use of default p-values is noted as a limitation and that this could introduce errors. |
| Fukuta (2016)^(1)^ | Exercise Training  (included: aerobic, combined) | VO_2peak_, 6MWT | Methods as stated in publication  *“All the included studies did not report the standard deviation of the change or the correlation of the pre and post measurements and did only the pre and post measurements. Accordingly, the correlation was conservatively set at 0.5 as previously reported”*  No details are provided of the number of studies for which the change SD was calculated or performance of any sensitivity analysis. |
| Ganga (2017) | Exercise Training  (included: aerobic, resistance, IMT) | VO_2peak_ | No clear details of methods for handling missing SDs are reported. |
| Giuliano (2017) | Resistance training | VO_2peak_, 6MWT | Methods as stated in publication  *“The standard deviation of the change score was calculated from the baseline and follow-up standard deviations by assuming that the correlation between baseline and follow-up scores was 0.8”*  No details are provided of the number of studies for which the change SD was calculated or performance of any sensitivity analysis. |
| Grosman-Rimon (2018) | Exercise Training  (included: aerobic, resistance, IMT) | VO_2peak_, 6MWT | SEs were converted to SDs, but no clear methods of handling missing change SDs are reported. |
| Gu (2017) | Tai Chi | 6MWT | Methods as stated in publication  *“If continuous data were expressed as median and interquartile range, the median was considered equivalent to the mean, and the relationship between the interquartile range and the standard deviation was approximately computed as SD = IQR/1.35”*  No details are provided of any other methods to be utilised to handle missing change SDs. The review notes one failure to acquire raw data, but does not provide details as to what data this was in relation to. |
| Ismail (2014) | Aerobic Training | VO_2peak_ | Methods as stated in publication  *“Revman 5.1 enabled calculation of post-intervention change from baseline for standard*  *deviation, using change in mean values, number of subjects and p-value or preferably 95% confidence intervals. In many cases where exact p-values were not provided, we used default values e.g. p <0.05 became p = 0.049”.*  No details are provided of the number of studies for which the change SD was calculated or performance of any sensitivity analysis. The use of default p-values is noted as a limitation and that this could introduce errors. |
| Jewiss (2016) | Resistance training | VO_2peak_, 6MWT | Methods as stated in publication  *“Change in post-intervention mean was calculated by subtracting baseline from post-intervention values. Data required was either (i) 95% confidence interval data for pre–post-intervention change for each group or when this was unavailable, (ii) actual p values for pre–post-intervention change for each group or if only the level of statistical significance was available, or (iii) we used default p values e.g. p< 0.05 becomes p = 0.049, p <0.01 becomes p=0.0099 and p=not significant becomes p=0.05*”.  No details are provided of the number of studies for which the change SD was calculated or performance of any sensitivity analysis. The use of default p-values is noted as a limitation and that this could introduce errors. |
| Montemezzo (2014) | Inspiratory Muscle Training (IMT) | VO_2peak_, 6MWT | Methods as stated in publication  “*When mean and SD required to perform the meta-analysis was not available in the original article, we contacted the authors to request additional information….. When the SD of change was not available, the SD of the baseline measure was used for the meta-analysis”*  No details of which studies the baseline SD was utilised for. |
| Neves (2014) | Neuromuscular Electrical Stimulation (NMES) | VO_2peak_, Peak Workload | Methods as stated in publication  *“The standard deviation was calculated for each study based on the change score method.*  *SD of each study calculated using change score method”*  The publication does not state the correlation value utilised or any associated sensitivity analysis. The review does note that if no SD is available to calculate the change score SD the study was excluded. The review notes the SD was not given in 2 studies; one was extracted from visual analyses and one was excluded. These studies are referenced and identifiable. |
| Neto (2018) | Aerobic Training | VO_2peak_ | Methods as stated in publication  *“Conversion of nonparametric data to means and standard deviation (SD) was based on recently*  *established methods (Wan et al. 2014). When the SD of change was not available, but confidence interval was available, we converted to SD as guidance by Higgins and Green”* |
| Neto (2016)a | Neuromuscular Electrical Stimulation (NMES) | VO_2peak_, 6MWT | No details provided of any methods to handle missing change SDs and no mention of any missing SD. |
| Neto (2016)b | Combined (endurance) training and Inspiratory Muscle Training (IMT) | VO_2peak,_ Exercise Time | No details provided of any methods to handle missing change SDs and no mention of any missing SD. |
| Neto (2015) | Hydrotherapy | VO_2peak_, 6MWT | No details provided of any methods to handle missing change SDs and no mention of any missing SD.  . |
| Neto (2014) | Yoga | VO_2peak_ | No details provided of any methods to handle missing change SDs and no mention of any missing SD. |
| Pandey (2015) | Exercise training  (included: aerobic, combined) | VO_2peak_ | No details provided of any methods to handle missing change SDs and no mention of any missing SD. |
| Ren (2017) | Tai Chi | VO_2peak_, 6MWT | Methods as stated in publication  *“…one study did not report the SDs of the mean, we thus used t-values to calculate the SDs…..*  *One article provided the median change (first quartile, third quartile), so we used the formula to estimate the mean changes and SDs”*  The specific studies to which these methods were applied are not identified and the formula referred to is not provided. |
| Santos (2018) | Aerobic, Resistance, Combined training | VO_2peak_ | No specific details provided of any methods to handle missing change SD and no mention of missing SD. Papers were to be excluded if there was absence of both means and SD.  Methods as stated in publication  *“In one study, the data was described in median and interquartile range and not in mean and standard deviation. Thus, we converted the median and interquartile range to obtain a good*  *estimate of the mean and standard deviation of the sample using the following methodology: Through the median values and interquartile ranges the percentile values were estimated from*  *5 to 95%, for every 5%, by proportional estimates. Using the estimated values, fourth order polynomial equations were calculated with good linearity (r2 > 0.9). The four polynomial equations (one for each sample) were obtained according to each sample size, as previously described. Finally, with the projection of the individual those values average and the standard deviation values were calculated”*  The study with median data which was converted is identified. |
| Vromen (2016) | Aerobic training | VO_2peak_ | No details are provided in methods section of how missing change SDs will be handled, however, in the limitations section it is noted that SD of change was missing for several studies and that a correlation of 0.7 was used.  No details are provided of which studies this was applied to. No details of any associated sensitivity analysis. |
| Zhang (2016) | Exercise training  (included: aerobic, resistance, combined, tai Chi, yoga) | VO_2peak_ | No details provided of any methods to handle missing change SDs and no mention of missing SD. The analysis method appears to be a comparison of pre-post change in the experimental groups and not a difference between control and exercise group, and no mention of any possible missing follow-up SDs or any approach to deal with. |

1. Analysis also included effect of drug trials, but exercise intervention analysis presented separately. MA: meta-analysis, SD: standard deviation, SE: standard error, SR: systematic review
