# Supplementary material for: Reported methods for handling missing change standard deviations in meta-analyses of exercise therapy interventions in patients with heart failure: A systematic review
Source: PLoS One. 2018 Oct 18;13(10):e0205952. doi: 10.1371/journal.pone.0205952 (PMC6193694; doi:10.1371/journal.pone.0205952)
Supplement: S3 Table — (DOCX) [file pone.0205952.s004.docx]

**S3 Table** Additional details of included meta-analyses

| **Study** | **Population** | **Study Designs Included** | **End Search Date** | **Summary Statistical Methods**  **for exercise capacity** | **Number of studies included in MA of Exercise Capacity** |
| --- | --- | --- | --- | --- | --- |
| Adsett (2015) | HFrEF | RCTs, Controlled, Single Group Studies | March 2014 | CMA  Hedges g for mean difference between change in groups  Random Effects | VO_2peak_ = 3 (4 included SR, but insufficient raw data of one study for MA)  6MWT = 2 (4 included SR, but insufficient raw data of one study for MA)  Peak Power = 3 |
| Chan (2016) | HFpEF | RCT | September 2015 | Revman  MD change pre-post between groups  Fixed Effects | VO_2peak_ = 5  6MWT = 5 |
| Dieberg (2015) | HFpEF | RCT | October 2014 | Revman  MD change pre-post between groups  Fixed Effects | VO_2peak_ = 5  6MWT = 5 |
| Fukuta (2014) | HFpEF | RCT | June 2014 | CMA  MD change pre-post between groups  Fixed Effects | VO_2peak_ = 4  6MWT = 4 |
| Ganga (2017) | HF with LVAD | RCTs, & Observational | Dec 2015 | CMA  SMD change pre-post between groups | VO_2peak_ = 4  (only RCTs included in meta-analysis) |
| Giuliano (2017) | HFrEF | RCT & Controlled | 10 July 2016 | Stata  MD change pre-post between groups  Fixed Effects | VO_2peak_ = 9  6MWT = 4 |
| Grosman-Rimon (2018) | HF with VAD | RCTs, Quasi & Observational | Nov 2015 | Revman  MD change pre-post between groups  Random Effects | VO_2peak_ = 4  6MWT =3  (only RCTs included in meta-analysis) |
| Gu (2017) | HF | RCT | 2 June 2016 | Revman & Stata  MD change pre-post between groups  Random Effects | 6MWT = 10 |
| Ismail (2014) | HFrEF | RCT | 2012 | Revman  MD change pre-post between groups  Random Effects | VO_2peak_ = 3 (High intensity)  VO_2peak_ = 29 (Vigorous intensity)  VO_2peak_ = 20 (Moderate intensity)  VO_2peak_ = 2 (Low intensity) |
| Jewiss (2016) | HFrEF | RCT | May 2016 | Revman  MD change pre-post between groups  Random Effects | VO_2peak_ = 10 (combined)  VO_2peak_ = 4 (resistance)  6MWT = 7 (combined)  6MWT = 2 (resistance) |
| Montemezzo (2014) | HF | RCT | August 2013 | Revman & CMA  MD change pre-post between groups  Random Effects | VO_2peak_ =4  6MWT = 4 |
| Neves (2014) | HFrEF | RCT | March 2014 | Revman & CMA  MD change pre-post between groups  Random Effects | VO_2peak_ = 7 (NMES v. Exercise)  Peak Workload = 2 (NMES vs. exercise)  VO_2peak_ = 9 (NMES v. Control)  Peak Workload = 3 (NMES vs. Control) |
| Neto (2018) | HFrEF | RCT | October 2017 | Revman  MD change pre-post between groups  Random & Fixed Effects | VO_2peak_ = 13 |
| Neto (2016)a | HF | RCT | May 2014 | Revman  MD change pre-post between groups  Random Effects | VO_2peak_ = 6 (NMES vs. Aerobic)  VO_2peak_ = 3 (NMES vs. Control)  6MWT = 5 (NMES vs. Aerobic)  6MWT = 6 (NMES vs. Control) |
| Neto (2016)b | HF | RCT | April 2015 | Revman  MD change pre-post between groups  Fixed Effects | VO_2peak_ = 3  Exercise Time = 2 |
| Neto (2015) | HF | RCT | May 2014 | Revman  MD change pre-post between groups  Random & Fixed Effects | VO_2peak_ = 2 (Hydro vs. Control)  VO_2peak_ = 2 (Hydro vs. Aerobic)  6MWT = 2 (Hydro vs. Control) |
| Neto (2014)a | HF | RCT | December 2013 | Revman  MD change pre-post between groups  Fixed Effects | VO_2peak_ = 2 |
| Pandey (2015) | HFpEF | RCT | NR | Stata (Metan & Metareg) & Revman  WMD change from baseline between groups  Random Effects | VO_2peak_ = 4 |
| Ren (2017) | HF | RCT | 16 Sep 2017 | Revman & Stata  MD change pre-post between groups  Random Effects | VO_2peak_ = 3  6MWT = 7 |
| Santos (2018) | HFrEF | RCT | March 2016 | Revman  MD change pre-post between groups  Random Effects | VO_2peak_ = 46 (Exercise vs. Control)  VO_2peak_ = 8 (Combined vs. Aerobic)  VO_2peak_ = 3 (Resistance vs. Aerobic) |
| Vromen (2016) | HFrEF | RCT | 1^st^ April 2015 | WMD for change pre-post between groups  Random Effects | VO_2peak_ = 17 |
| Zhang (2016) | HF | RCT | 2014 | Revman & Stata  MD of pre-post values for HF exercise group only  Random Effects | VO_2peak_ = 20 |

CMA: comprehensive meta-analysis, HF: heart failure, HFpEF: heart failure preserved ejection fraction, HFrEF: heart failure reduced ejection fraction, Hydro: hydrotherapy, RCT: randomised controlled trial, MA: meta-analysis, MD: mean difference, NMES: neuromuscular electrical stimulation, NR: not reported, SR: systematic review, VO_2peak_: maximal oxygen uptake, WMD: weighted mean difference, 6MWT: six minute walk test
